# Chronicity, self care, social and family support: how the patient has?

**DOI:** 10.1186/1758-5996-7-S1-A202

**Published:** 2015-11-11

**Authors:** Francisca Alexandra Araújo da Silva, Thereza Maria Magalhães Moreira, Luciana Gomes Catunda Menezes

**Affiliations:** 1 Universidade Estadual do Ceará, Fortaleza, Brazil

## Background

Diabetes mellitus is a non-communicable chronic systemic disease. It is estimated that in 2030 will have over 300 million diabetics worldwide. Perform preventive measures on the glycemic control, foot care and other education initiatives in diabetes favors the reduction of signs and symptoms of the disease progression.

## Objective

This study aimed to evaluate sociodemographic and clinical characteristics of a group of diabetics as well as meet social relations that permeate the self-care of these patients.

## Materials and methods

Cross-sectional study, epidemiological performed in a referral center for diabetes and hypertension the northeast region of Brazil in the period June to August 2013.

## Results

A total of 538 people with diabetes, and the study population was predominantly female (63 4%), married people or with fixed partners (55.6%), older (59.7%), Catholic (72.1%), low education, less than 10 yrs. (74%); 65% with incomes below twice the minimum wage. On clinical data 79.2% were hypertensive, 69.1% with over 10 yrs. of diagnosis of diabetes mellitus, 40.7% used medication by mouth for diabetes control, when asked about the help or assistance of others towards treatment, 62.3% reported not having social or family support or even encouragement to move with adherence to medication or non-medication treatment for diabetes. When asked about following the prescribed diet, only 60.4% reported following the diet routinely, as physical activity, 61.2% of persons were sedentary, 72.7% presented themselves with excess weight, and those with overweight or obese, 63.6% of diabetic people found themselves with altered glycemia, with greater 140 mg/dL at the time of data collection at random.

## Conclusion

Notoriously we see, when we analyze the data, the chronicity characteristics of the disease, lack of social and family support to these patients, worsening of clinical status and poor adherence to self-care of the person with Diabetes Mellitus. It is extremely important that multidisciplinary actions aimed at intensive care to this population are drawn at national level through effective public health policies they can minimize grievances as well, decrease complications using lines of care in diabetes care and management.

**Figure 1:**
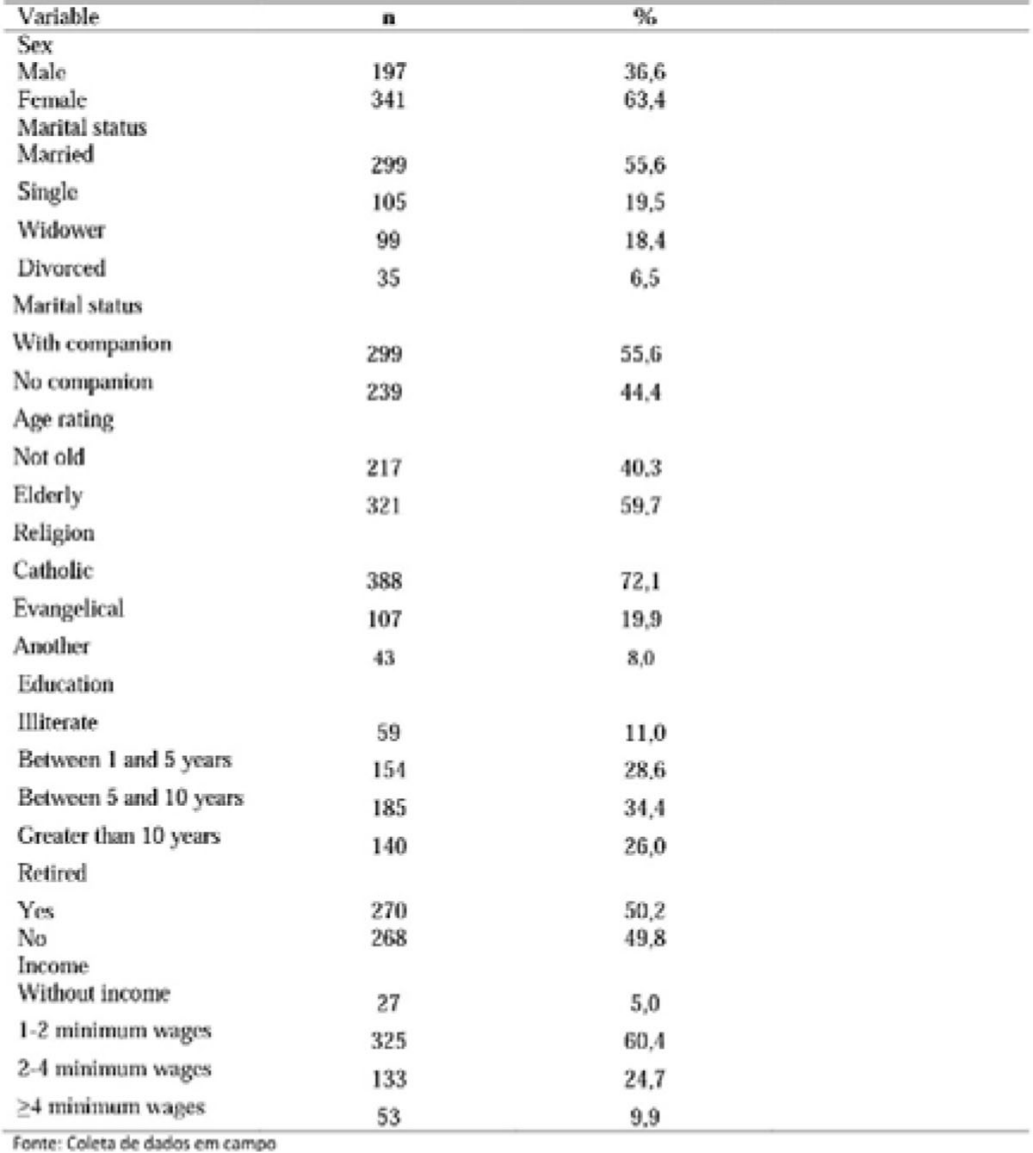
Socio-demographic characteristics of people with diabetes mellitus treated as a referral center in a Northeastern capital. 2015.

**Figure 2:**
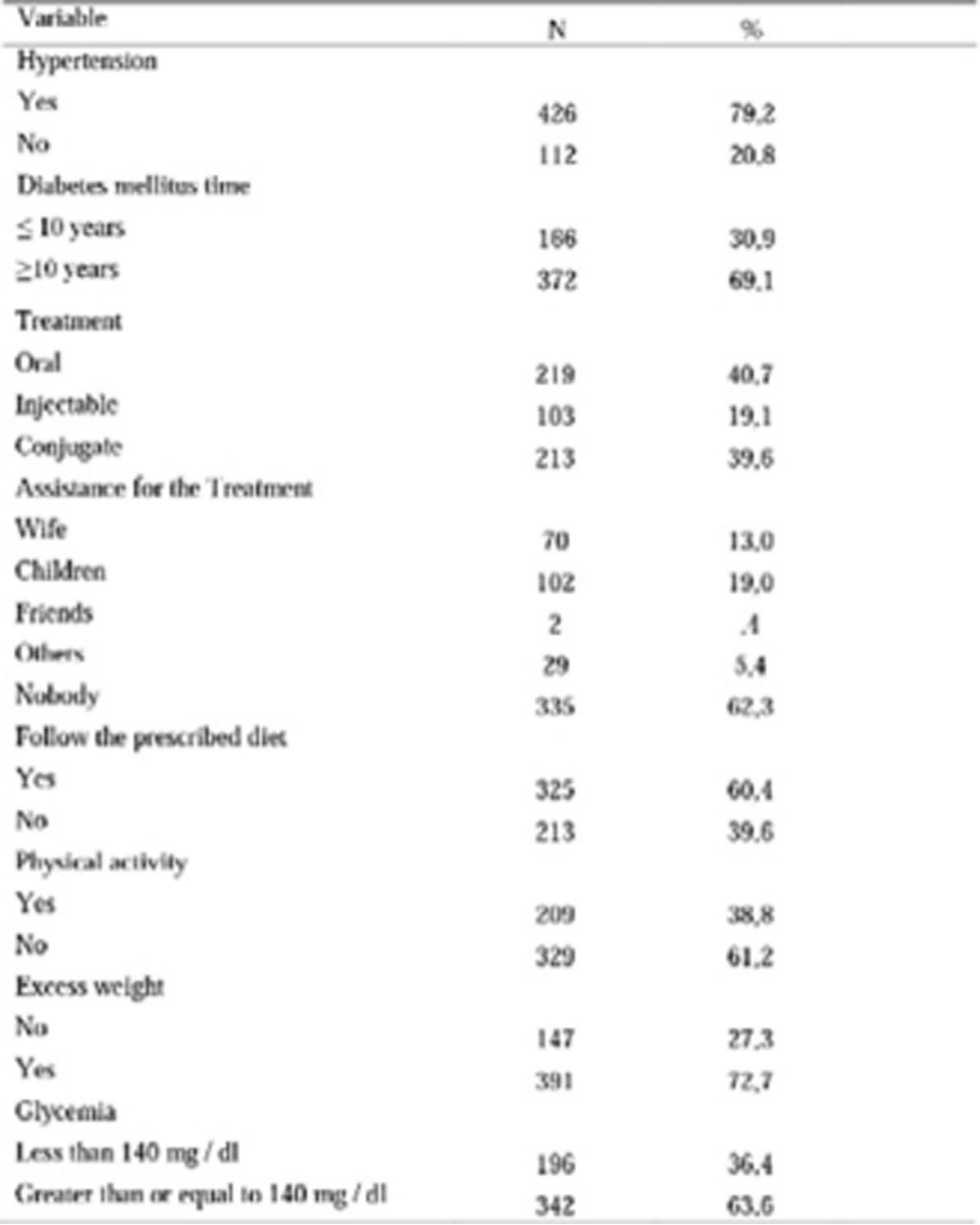
Clinical date of people with diabetes mellitus treated as a referral center in a Northeastern capital. 2015.

